# Research on Early Warning Models for Swine Feeding Dynamic Signatures Based on Electronic Automated Feeding Data

**DOI:** 10.3390/ani16121880

**Published:** 2026-06-17

**Authors:** Yima Wang, Yuancheng Xie, Jianlan Wang, Yuhan Zhang, Wei Wei, Jie Chen, Jinbi Zhang, Zengxiang Pan

**Affiliations:** 1 College of Sciences, Nanjing Agricultural University, Nanjing 210095, China; 23123202@stu.njau.edu.cn (Y.W.); 23123217@stu.njau.edu.cn (Y.Z.); 2 College of Artificial Intelligence, Nanjing Agricultural University, Nanjing 210095, China; xieych@njau.edu.cn; 3 Shiji Biotechnology Co., Ltd., Hefei 230091, China; wjianlan2022@126.com; 4 College of Animal Science & Technology, Nanjing Agricultural University, Nanjing 210095, China; wei-wei-4213@njau.edu.cn (W.W.); jiechen@njau.edu.cn (J.C.); 5 College of Animal Science and Technology, Jinling Institute of Technology, Nanjing 211169, China; zhangjinbi@jit.edu.cn

**Keywords:** precision livestock farming, electronic feeding station, feeding dynamic signatures, early warning system, XGBoost, LightGBM, machine learning

## Abstract

One of the keys to improving feed conversion rates in Precision Livestock Farming (PLF) is the early identification of growth impediments. However, the swine farming data collected by Electronic Feeding Station (EFS) are often disorganized and lack effective labeling. Data from healthy pigs are frequently intermixed with that from sick pigs, leading to label leakage and survivor bias in models, particularly when age is included as a feature. To address these known issues, this study breaks away from traditional modeling methods. First, we clean and classify the time-series data from electronic feeding stations, using age-cohort baselines as one of the criteria for determining high and low productivity, thereby avoiding problems such as label leakage. Next, we construct a high-dimensional feature matrix that captures dynamic derivatives such as feeding acceleration and weight gain acceleration, which together serve as behavioral feature fingerprints. To test the system, we optimized the mixed-model algorithm and evaluated the model based on behavioral deviations among individual pigs after removing all absolute age labels. Our results indicate that the full-feature model achieved an ROC-AUC of 0.778 and an F1-score of 0.4137 at the optimal threshold. Interestingly, SHAP attribution analysis revealed that “intake peer deviation,” “Cumulative Intake and Lifetime Avg Intake,” and “feeding acceleration” served as precursors to low productivity and growth retardation in this dataset, with these factors proving more significant than absolute feed intake or age. Our ablation experiments confirmed that a model based solely on behavioral features (excluding age labels) maintained an ROC-AUC of 0.773, successfully decoupling pig growth performance from growth stage. Our model can detect changes in feeding dynamic signatures at an average of 12.3 days, thereby providing insights for pig growth assessment, health monitoring, or more informed culling decisions.

## Simple Summary

In modern swine farming, identifying the early symptoms and characteristics of low-performing pigs (referred to in this paper as “underperforming pigs”) and using this information for prevention pose a significant challenge. This study proposes a machine learning method designed to utilize automated data from electronic feeding stations to provide early health alerts for pigs. Unlike traditional methods that focus solely on monitoring decreases in feed intake, we analyzed dynamic behavioral patterns, such as feeding acceleration and the rate of empty visits—that is, when pigs enter the feeding station but do not eat. To account for biological variation across growth stages, we compared each pig’s behavior with that of its age-matched peers to establish a baseline for that age group, which served as one of the criteria for assessment. The results indicate that our study can detect these subtle behavioral changes and issue early warnings an average of 12.3 days before pigs exhibit reduced productivity or production issues. The methodology used to construct this model can help farmers identify “underperforming pigs” early, thereby reducing feed waste while improving economic efficiency and animal welfare.

## 1. Introduction

In today’s intensive electronic pig farming industry, Precision Livestock Farming (PLF) has become widespread and serves as a key means of improving farming efficiency [1]. Although the use of electronic feeding stations provides a continuous stream of data on individual feeding behavior and body weight changes, these data often suffer from various shortcomings. For instance, it frequently contains a significant amount of random noise and erroneous data [2,3]. Additionally, since pigs at different growth stages are mixed together, it is difficult to accurately assess their growth efficiency and actual physiological condition. Extracting biologically meaningful and actionable signals from such noise-laden data and converting them into predictive indicators for growth retardation remains a significant challenge. At the same time, some studies point out that a significant gap in current research is the lack of techniques for assessing emotional states (including both positive and negative states) and social states [4,5].

Traditional assessments of growth performance primarily rely on manual observations or retrospective weight measurements. This approach is not only time-consuming and labor-intensive but also suffers from significant time lags. By the time pigs exhibit significant weight loss or slow growth, their physiological functions have often already suffered irreversible damage, which can directly lead to reduced farming profits and wasted resources [6]. Although previous studies have attempted to develop predictive models using machine learning techniques, two critical bottlenecks remain to be addressed in real-world commercial farming environments.

First, existing data and training methods can lead to label leakage and “data end confusion”—that is, we often cannot determine what a sudden break in the data signifies. It could represent the normal market-ready slaughter of healthy pigs, culling due to disease, or transfers between different farming operations. Additionally, models frequently rely on absolute age-based features, leading to path dependence or severe label leakage—specifically, an over-reliance on strong correlations between age and growth performance. These situations often yield seemingly correct inferences, but in reality, the conclusions are not derived from the pigs’ behavioral characteristics.

Second, existing models and their predictive results suffer from significant lag, making it difficult to achieve real-time responses and dynamic adjustments during the farming process, which limits their utility in supporting production decisions. This is particularly true for non-visual, entry-level personalized pig farming (PLF) devices. Due to the lack of efficient data processing and real-time feedback mechanisms, such devices are mostly used for post-production data review and record-keeping, rather than being truly integrated into daily production management processes to provide real-time decision support for critical aspects such as feeding strategies, health alerts, or environmental control. Consequently, although these devices have improved the traceability of farming data to some extent, their capabilities in enhancing farming efficiency and responsiveness remain inadequate, deviating from the original intent of PLF technology to optimize real-time decision-making [7].

To address these issues and identify correlations between behavioral characteristics and growth performance, we propose a machine learning early warning method based on feeding dynamic signatures. Our goal is to establish a model system capable of predicting growth risks based on short-term relative behavioral deviations while maintaining a certain degree of independence from absolute age. The main contributions of this paper include:

1. We propose a multi-criteria outlier detection method to clean the data, removing noise without losing the characteristics of problematic pigs.
2. We propose a classification mechanism for obtaining “low-productivity period slices,” converting “underperforming pig” samples into “underperforming pig period” samples to acquire more anomalous data. By decoupling age from relative growth performance, we address the challenges of sparse, fragmented, and hard-to-obtain samples.
3. We construct a behavioral profile database that includes nighttime feeding patterns, empty feeding station visit rates, and age-related deviations, while simultaneously building an integrated learning model for prediction and evaluation.
4. We perform an in-depth decomposition of our hybrid model using the SHAP interpretability framework to quantify the contribution of feeding dynamic signatures across different physiological stages, thereby opening the model’s “black box” from a data science perspective.

## 2. Materials and Methods

### 2.1. Data Acquisition and Cohort Characteristics

**Figure 1:**
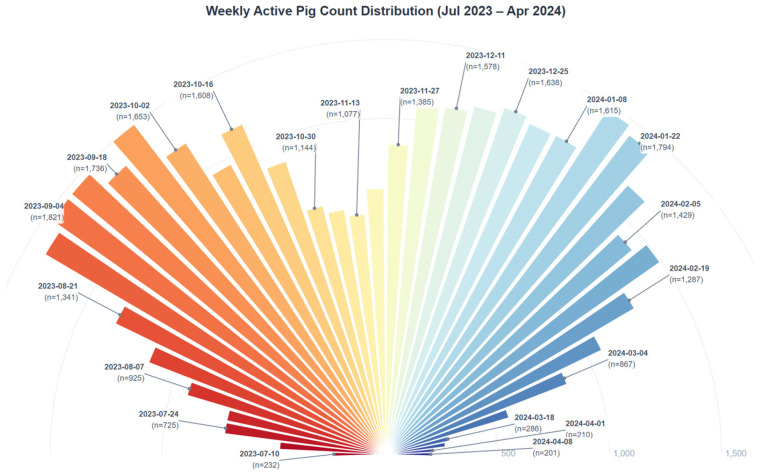
Weekly distribution of active pigs recorded by the Intelligent Feeding System from July 2023 to April 2024. Each radial bar represents one calendar week, with bar length indicating the number of distinct pigs detected. Peak activity was observed during the September–October and December–January periods (*n* ≈ 1600–1850 pigs/week), with a gradual decline toward the end of the trial in April 2024. The color gradient is for visual clarity and does not represent distinct variables.

The dataset used in this study originated from the core breeding farm of Shiji Biotechnology Co., Ltd. (Maanshan, China), a large pig breeding enterprise in China. The pig feeding intake phenotypic data were recorded by using 9ZC-170 Intelligent Feeding System (IFS, Guangdong Guangxing Poultry Equipment Co., Ltd., Guangzhou, China), covering the entire feeding process of various pig breeds from 2023 to 2024. The initial dataset included 6035 unique pigs, primarily including five breeds: Duroc (DR; *n* = 2274), Landrace (LR; *n* = 1544), Large White (LW; *n* = 1149), Pietrain (PI; *n* = 1058), and Pietrain × Duroc (PX; *n* = 10). The raw automatic feeding database contained 4,254,016 records. Each record comprises information related to the feeding station (station ID), individual animal identification (e.g., RFID tag, ear tag, and unique individual ID), temporal data (entry and exit timestamps), and feeding performance metrics (e.g., feeding duration, feed intake, and weight measurements). The weekly distribution of active pigs recorded by the Intelligent Feeding System is shown in Figure 1. Additionally, metadata including breed, birth date, gender, and age were recorded for each pig. Detailed descriptions of all variables are provided in Appendix A (Table A2).

**Figure A1:**
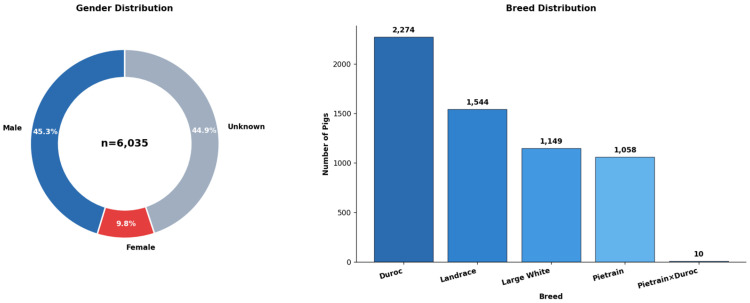
Distribution of experimental pig population by gender (**left**) and breed (**right**). The donut chart shows the proportions of male (45.3%), unknown (44.9%), and female (9.8%) individuals across 6035 unique pigs. The bar chart illustrates the count of pigs by breed, with Duroc being the most represented breed (*n* = 2274), followed by Landrace (*n* = 1544), Large White (*n* = 1149), Pietrain (*n* = 1058), and Pietrain × Duroc (*n* = 10).

It is important to note that due to the significant imbalance in sex ratios within actual production batches (e.g., the disparity between barrows and gilts), the gender variable was intentionally excluded during the feature engineering phase. As illustrated in the Appendix A (Figure A1) and the corresponding statistical distribution in Table A1, this imbalance could lead to dimensional bias in the machine learning model’s tree-splitting process. By excluding this variable, the research focuses exclusively on mining temporal behavioral patterns and growth dynamics across the diverse breed population.

### 2.2. Data Preprocessing and Quality Control

When automatic feeders collect pig feeding data, the resulting raw time-series data often contain significant noise or local anomalies due to missing or mislabeled tags, sensor errors, and low robustness of the data collection process. Examples include completely implausible weight values or single-point outliers (e.g., ≥1000 kg or certain negative values), obvious weight drift (where weight values suddenly change by an order of magnitude but the overall trend remains largely unchanged), and empty tag information (e.g., empty RFID tags or bit errors). In such animal behavioral and agricultural big data research, ensuring the quality of our datasets is a prerequisite for obtaining reliable machine learning models and their predictions. This study employs a multi-step approach to construct an automated data cleaning workflow that removes significant noise from the data while preserving the behavioral characteristics of the pigs, ensuring that anomalous features remain detectable rather than being erased due to excessive data cleaning [8].

#### 2.2.1. Data Standardization and Bottom-Level Cleaning

First, we consolidated the EFS’s raw Excel spreadsheet files into a single CSV file, performed a full-field scan to remove zero-width characters, and carried out multi-space compression and type matching. To address missing and erroneous tags, we prioritized linking ear tags with RFID tags through multi-field matching. Additionally, we employed median-based dynamic filtering to handle abnormal weight fluctuations and clearly unreasonable data. Previous studies have shown that the natural daily fluctuation range for pig weight typically falls between 0.9 kg and 1.4 kg. In our study, this range was used as a physiological reference baseline, though not as a strict cutoff. By performing an average analysis on this broad sample, we established a horizontal benchmark for pigs at this age. Regarding individual pig timelines, while rapid weight jumps (e.g., weight increases exceeding 5 kg between two readings within a few hours) were excluded as sensor errors or crowding incidents (where more than one pig entered the weighing area or foreign objects were present), we employed a second-derivative algorithm to examine the rate of change in the data. Concurrently, using a windowing method, we establish horizontal windows within the data to examine fluctuations within each window, ensuring that partial smooth variations in weight changes exceeding 1.4 kg or falling below 0.9 kg are retained as features rather than discarded as noise. This cleaning method ensures that the feeding dynamic signatures and physiological characteristics of underperforming pigs are preserved, while preventing errors or erroneous data from entering the model’s training and leading to incorrect results.

#### 2.2.2. Multi-Criteria Outlier Detection

To address noise in data captured by automatic feeding systems (AFSs), this study designed and implemented three collaborative classification methods to identify and exclude outlier data:

- Median- and range-based rules: Automatic weighing systems are prone to measurement errors caused by herd movement or foreign objects. Influenced by defecation, feeding, and water intake, the natural daily variation in pig body weight typically ranges from 0.9 kg to 1.4 kg. This daily variation must be taken into account. We first calculated the median body weight data for the same pigs over a past period to serve as a baseline for pigs within that age group. The values of −0.9 and +1.4 relative to this baseline are used as thresholds. When the variation between adjacent valid observations significantly exceeded (e.g., by several times) these physiological limits, and if the time-series weight data reveals clearly anomalous values, the system classifies them as “out-of-range data” and excludes these anomalies to prevent the algorithm from overfitting to distorted data. Conversely, if the deviation from the baseline is small and the change is relatively stable (i.e., not a sudden surge or plunge), the data is considered a valid feature and retained.
- Jump-based Rule: To reasonably handle certain extreme data points (where some noise data consisted of single points or a small number of consecutive points exhibiting sudden, drastic changes), this study employs the interquartile range (IQR) and rolling median to perform joint smoothing detection on individual growth curves. Nonparametric estimators based on the median often exhibit strong robustness to outliers [9]. Therefore, for an individual’s weight data, we establish a sliding window of a fixed duration and use the local median within the sliding window as a reference benchmark, calculating relative and absolute deviations to address noise from abrupt changes.
- Isolated Outlier Diagnosis: To address common sensor resets or brief physical disturbances in time-series data, a specialized “pattern recognition” mechanism was designed to accurately filter out spike–drop pairs (sequences in the dataset such as normal value–0–normal value or normal value–extreme value–normal value). When an observation experiences a sharp rise or sudden drop, followed immediately by a rapid return to within the confidence interval, it is identified as typical isolated impulse noise, and the peak/trough is excluded.

During the preprocessing phase, anomalous records involving erratic feed intake, extreme weight fluctuations, and logical inconsistencies were excluded. After stringent quality control, 3,869,423 valid records remained for analysis. Through the aforementioned dual filtering based on robust statistics and biological rules, the system effectively ensures the smoothness and monotonicity of the retained data, establishing a high-quality foundation for subsequent feature extraction.

### 2.3. Data Analysis Methods and Models

#### 2.3.1. Weight Trajectory Smoothing via Legendre Series

After outliers are removed, the remaining discrete values are inevitably affected by high-frequency measurement fluctuations or missing data. To extract high-quality growth features, directly applying finite difference derivatives to the discrete points would significantly amplify the residual noise. Therefore, this study introduces a Legendre series expansion to perform global quadratic smoothing and continuous reconstruction of the body weight trajectories [10].

Drawing on the strategy proposed by Aghili et al. [11], this study treats the discrete weight observation sequence of pigs as a continuous function of time. We use orthogonal Legendre polynomials to approximate this function [12]. To satisfy the domain requirements for the orthogonality of Legendre polynomials, the actual growth monitoring time t must be linearly mapped to the standard interval $\left[-1,1\right]$:

(1) $$\tau =\frac{2t-\left(t_{max}+t_{min}\right)}{t_{max}-t_{min}}$$

Subsequently, the swine weight trajectory can be approximated by the partial sum of the first n orders of the Legendre series:

(2) $$W\left(\tau \right)\approx \sum_{n=0}^{N}C_{n}P_{n}\left(\tau \right)$$

(Note: $C_{n}$ represents the coefficients for the corresponding orders, obtained by minimizing the sum of squared errors (SSE) through numerical integration).

Distinguishing it from traditional least-squares polynomial fitting, the coefficients in this method are derived directly through numerical integration, effectively avoiding Runge’s phenomenon and the issues associated with ill-conditioned matrix inversion commonly found in high-order fitting. By optimizing the order to minimize the Sum of Squared Errors (SSE), the algorithm adaptively filters high-order stochastic noise while fully preserving the actual biological growth trends [13]. More importantly, by utilizing the analytical form of this function, the system can directly calculate the continuous instantaneous weight change rate through the analytical derivative of the Legendre polynomials, completely bypassing the oscillations caused by numerical differentiation [14,15]. The formula for instantaneous weight gain (analytical derivative) is:

(3) $$ADG\left(t\right)=\frac{dW\left(t\right)}{dt}=\frac{d\tau }{dt}\cdot \frac{dW\left(\tau \right)}{d\tau }=\frac{2}{t_{max}-t_{min}}\sum_{n=1}^{N}C_{n}P_{n}^{\prime }\left(\tau \right)$$

#### 2.3.2. Ground Truth Labeling Strategy

To provide a rigorous binary target labels (Y = 0 or 1) for the predictive model within unsupervised production data, this study proposes an endpoint classification logic based on the growth impairment occurring within the next 7~14 days. For any observation day (Day t) in the life cycle, an individual is labeled as a high-risk growth-impaired sample (Y=1) if it satisfies any of the following conditions within a 7-day window:

1. The absolute increment of smoothed body weight over 7 consecutive days is less than the biological minimum threshold (e.g., ΔWeight < 0.5 kg, indicating that the individual has entered a state of substantial physiological stagnation.)
2. Data for the individual is permanently interrupted, and the absolute age at the time of interruption is strictly less than 140 days. This constraint effectively eliminates “survivor bias” from healthy individuals normally marketed after reaching target weight, ensuring the pathological nature of the disappearance event.
3. To account for absolute feed intake shifts caused by environmental factors (such as temperature fluctuations or feed formula changes) and to avoid structural biases introduced by cross-sectional cohort comparisons, this study employs a purely self-referential longitudinal labeling strategy. For any given observation day t, we compare the individual’s average daily feed intake over the subsequent 3 days against its own historical baseline from the preceding 7 days. A sudden, drastic reduction in feed intake is a strong primary indicator of physiological distress, illness, or severe growth retardation. Specifically, an individual is marked as anomalous if its future 3-day average intake drops by more than 10% compared to its own past 7-day average:

(4) $$Intake_{future\_3d}<Intake_{past\_7d}\times 0.75$$

(Note: $Intake_{future\_3d}$ represents the average daily feed intake from day t+1 to t+3, and $Intake_{past\_7d}$ represents the baseline average daily feed intake from day t−6 to t).

This mathematical criterion focuses exclusively on the individual’s temporal dynamics rather than peer deviation. By utilizing a 10% self-referential decline threshold, the model effectively filters out normal daily behavioral noise while isolating acute physiological anomalies. Furthermore, this pure longitudinal approach completely eliminates the risk of circular dependency between the labeling criteria and the cohort-based features used later in model training.

The above labeling procedure is applied within a 7-day window, specifically during the 7-day observation period. Once these observation windows are labeled, they are not relabeled; that is, none of the growth impairment labels overlap. We extract all such 7-day time slices, each containing a multidimensional feature matrices for that time period, to form the positive sample set. The negative sample set is derived from multidimensional matrices composed of non-overlapping data slices (also 7 days in duration) from other time periods.

#### 2.3.3. Feeding Dynamic Signatures

To distinguish individual physiological abnormalities from system-wide environmental fluctuations and inherent breed differences, this study established a cohort baseline. For any given observation day, this baseline was calculated as the average performance of all individuals of the same breed and age. Consequently, absolute feed intake was converted into the age-matched feed-intake deviation feature. This mathematical transformation effectively filters out collective false positives caused by environmental changes.

Additionally, the system extracts key feeding dynamics features to capture subtle behavioral abnormalities:

Night Feeding Ratio: The proportion of station visits between 22:00 and 04:00 the following day, used to quantify rhythm disturbances caused by social competition pressure or daytime feeding difficulties [16,17].

Empty Visit Ratio: The proportion of station entry events with near-zero actual feed intake, serving as a pathological indicator of ingestion disorders or restless behavior.

These feeding dynamic signatures, calibrated against the cohort baseline, were used directly as the high-dimensional feature input matrix X. Similarly, we computed second-order dynamic features of some feeding-related data, which will also be used as input features.

### 2.4. Ensemble Learning Framework (XGBoost and LightGBM)

After constructing a multidimensional feature library containing a large number of time slices and lifetime feeding behaviors, we built an early health warning classifier using an ensemble learning framework (combining XGBoost and LightGBM).

To address the issue of sample imbalance (the ratio of positive to negative samples is approximately 1:9, with a positive rate of 10.9%), we introduced a dynamic weight adjustment parameter, ‘scale_pos_weight’, into the loss function. By calculating the real-time ratio of negative to positive samples, we assigned a higher gradient gain penalty to the minority class (i.e., growth-restricted individuals). This rebalancing strategy forces the model to focus on capturing subtle behavioral anomaly signals during tree splitting, rather than falling into “shortcut predictions” for the majority class, thereby significantly enhancing the ability to identify potential “underperforming pigs” [18].

Furthermore, to accommodate the nonlinear temporal characteristics of pig feeding data, our model incorporates second-order dynamic features—including feeding rate and feeding acceleration—via a high-performance analysis engine based on the DuckDB database [19,20]. This shift from static to dynamic variables in data mining aims to capture specific changes in the slopes of temporal features when an individual transitions from a normal state to a “zombie pig” state. Furthermore, our model employs cumulative features (such as cumulative feed intake) and partial feeding dynamic signatures as surrogate variables for different growth stages. These behavioral indicators replace static variables such as absolute age and body weight, enabling the model to effectively avoid “shortcut learning” and focus on extracting features of pig behavior.

**Figure 2:**
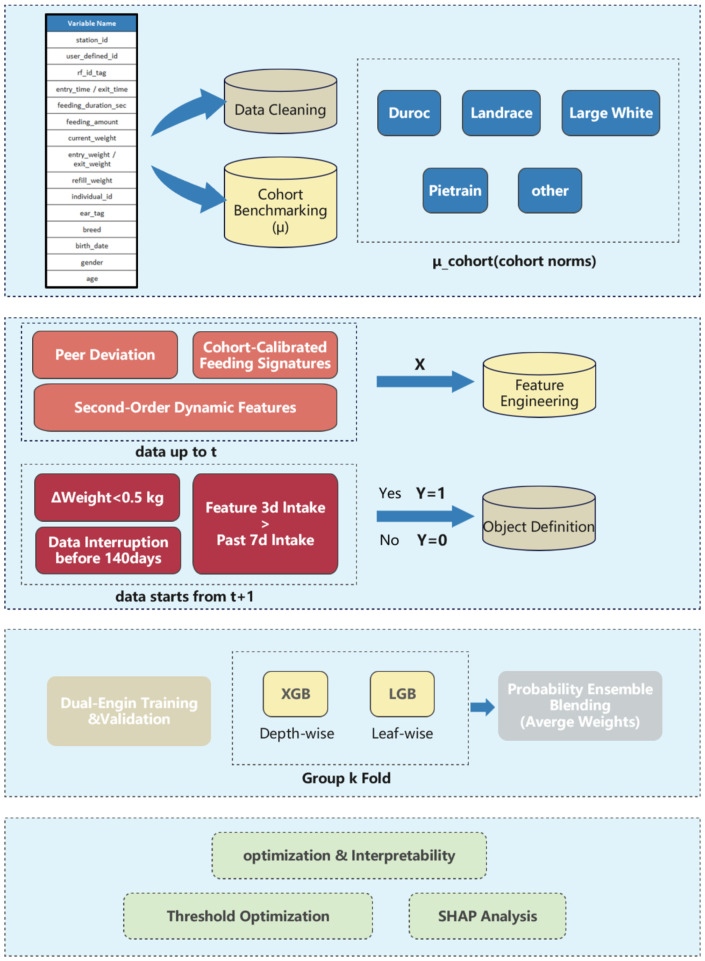
Model Flowchart. Describes the entire model workflow and data processing procedures.

Our model adopted simple probability averaging (also known as equal-weight blending), where the final probability is calculated as $P_{ensemble}=\left(P_{XGBoost}+P_{LightGBM}\right)/2$. The flowchart for this model is shown below (Figure 2).

### 2.5. Optimization and Robustness Strategies

To ensure the model’s generalization performance on independent individuals, the following strategies were implemented during the training phase:

#### 2.5.1. Bayesian Hyperparameter Optimization

This study utilized Bayesian optimization based on Tree-structured Parzen Estimator (TPE) to perform a global search of the hyperparameter space via the Optuna framework. Cooperative optimization focused on the learning rate (η), maximum tree depth, and subsampling ratio [21]. During 80 trials, the search intervals for Gamma (0.1–5.0) and Min_child_weight (5–30) were specifically expanded [22,23]. This approach aims to improve classification accuracy [24,25,26].

#### 2.5.2. Early Stopping and Overfitting Prevention

An Early Stopping logic was introduced. By monitoring PR-AUC fluctuations in real time on an independent internal validation set, training was halted and the model was rolled back to the optimal weights if performance does not significantly improve for 50 consecutive rounds. This effectively prevents the model from overfitting to sensor noise or specific growth patterns [27].

#### 2.5.3. Group-Based Validation (GroupKFold)

We performed rigorous GroupKFold cross-validation. This ensures that all historical records for a single pig (user_defined_id) exist only on one side of the partition (training set or test set) [28,29,30,31], thereby completely isolating the model from “data leakage” caused by individual characteristics and enabling a true assessment of predictive performance on the test set or for newly admitted pigs [32,33].

#### 2.5.4. Threshold Optimization via Validation Feedback

Given the scarcity of “zombie pig” samples (117,192:14,335; 8.18:1), this study abandoned static classification thresholds [34]. A dynamic threshold optimization mechanism was introduced in the validation set to find the cutoff point that maximizes the global F1 score by iterating over prediction probabilities. Here, we ultimately selected the optimal trade-off between “risk of missed detections” and “manual inspection cost” [35,36] Through an exhaustive search of the validation set, we precisely calibrated the optimal classification threshold to 0.383, which represents the maximum achievable F1 score in the current feature space: it does not result in excessive false positives but effectively identifies “underperforming pigs”.

### 2.6. Cross-Validation Strategy and Evaluation Metrics

To ensure rigorous evaluation and eliminate “Label Leakage,” this study utilized individual-based GroupKFold Cross-Validation (K=5).

Considering the imbalanced nature of the dataset, accuracy alone cannot objectively reflect the model’s interception capability. Thus, a multi-dimensional metric system derived from the confusion matrix was used, including Precision, Recall, and F1-Score [37]:

(5) $$Precision=\frac{TP}{TP+FP}$$

(6) $$Recall=\frac{TP}{TP+FN}$$

(7) $$F_{1}=2\times \frac{Precision\times Recall}{Precision+Recall}$$

In addition to global metrics, this study focuses on early warning time. By backtesting the model’s warning time against the actual time when individuals reached the health collapse threshold, we quantified the “golden window” available for clinical intervention [38].

### 2.7. SHAP-Based Interpretability and Ablation Study

In Precision Livestock Farming (PLF) applications, model interpretability is as critical as predictive accuracy. To address the “black-box” nature of models and identify the biological drivers behind growth collapse in pigs, this study introduces a game-theory-based attribution framework: SHAP (Shapley Additive exPlanations) [39,40,41,42].

To balance system-level performance evaluation with the tracing of underlying biological mechanisms, we innovatively designed a “Multi-scale Dual-track Interpretability Framework,” comprising the following two analytical dimensions:

- Macro-level Predictive Attribution (Full 24-dimensional Feature Set): In this dimension, SHAP is applied to the comprehensive 24-feature XGBoost model, which includes lifelong cumulative features, kinetic derivatives, and cohort deviations. The primary objective is to validate the effectiveness of our core engineering hypothesis—”lifelong physiological baseline + short-term cohort deviation”—and to quantify the model’s attention allocation across different time scales (short-term anomalies vs. long-term foundations) amidst complex production noise using SHAP Summary Plots [39].
- Micro-level Pathological Tracing (Focused Behavioral Slices): Given the high degree of multicollinearity and dimensional coupling in the 24-feature model, we extracted independent behavioral slices, such as “night feeding ratio” and “empty visit ratio,” for a secondary SHAP analysis to test specific ethological hypotheses. This dimension aims to isolate interference from complex kinetic features and directly quantify the marginal contributions of “pseudo-appetite” and “social marginalization” (bullying logic) to warning signals, providing underlying pathological and behavioral evidence for macro-level alerts.

Definition of SHAP Dependence Plots: Specifically, this study utilizes SHAP Summary Plots and SHAP Dependence Plots. Unlike traditional Partial Dependence Plots (PDP), which only display the global average marginal effect of a feature on predicted outcomes, the SHAP dependence plots employed here represent a method of “Aggregation of Local Explanations.” In these plots, each scatter point represents the Local SHAP Value of an individual observation. This instance-based projection method not only precisely delineates the non-linear mapping boundaries between a single feature (e.g., lifelong average daily intake) and predictive risk but also intuitively reveals Local Interaction Effects between that feature and other covariates (e.g., age) at the individual level through the vertical dispersion of attribution values. This approach effectively prevents the loss of heterogeneous information typically caused by global averaging.

To verify whether the feeding dynamic signatures extracted in this model possess generalization capabilities across different growth stages, we designed an abandonment study. After removing day-of-age (current_age) and absolute body weight features—which are prone to shortcut learning—the model was retrained independently using only the feeding dynamic signatures and relative behavioral deviation. This step was intended to demonstrate, from a fundamental perspective, that the dynamic changes in pigs’ feeding rhythms can achieve complete decoupling of static variables, thereby serving as a reliable basis for assessing pig growth.

## 3. Results

### 3.1. Model Performance and Ablation Analysis

The ensemble model (combining XGBoost and LightGBM) was evaluated using a strict individual-isolated 5-Fold GroupKFold cross-validation to eliminate data leakage across temporal sequence.

**Figure 3:**
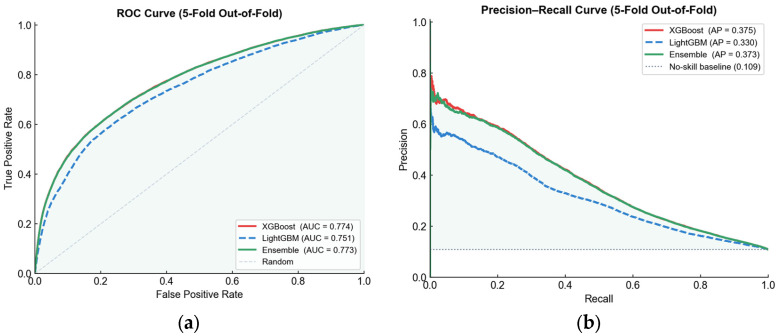
Classification performance evaluation of the health prediction models (5-Fold Out-of-Fold). (**a**) Receiver Operating Characteristic (ROC) curve; (**b**) Precision–Recall (PR) curve.

**Figure 4:**
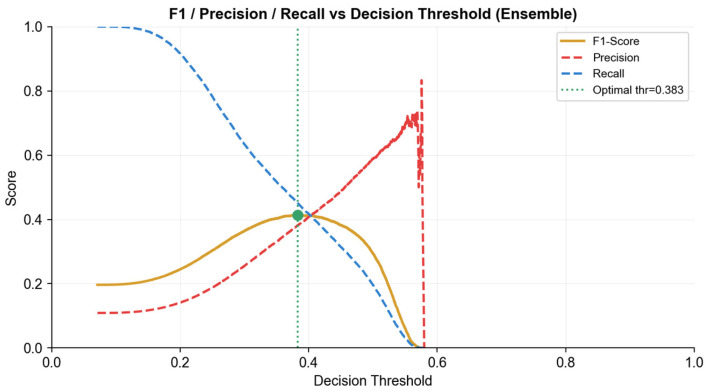
Dynamic Threshold Optimization.

The overall model performance is shown in Figure 3a,b. Given the severe class imbalance in this scenario (where positive anomaly events accounted for approximately 10.9%), the system demonstrated acceptable performance. Under cross-validation, the full-feature ensemble model achieved an ROC-AUC of 0.778 and a PR-AUC of 0.381 (significantly outperforming the no-skill baseline of 0.109). As depicted in the dynamic threshold feedback curve (Figure 4), at the optimally calibrated decision threshold (0.383), the model’s F1-Score reached 0.4137, with a Precision of 38.16% and a Recall of 45.17%. These metrics indicate that the model retains a certain ability to detect abnormal physiological states even in environments with highly imbalanced samples.

**Figure 5:**
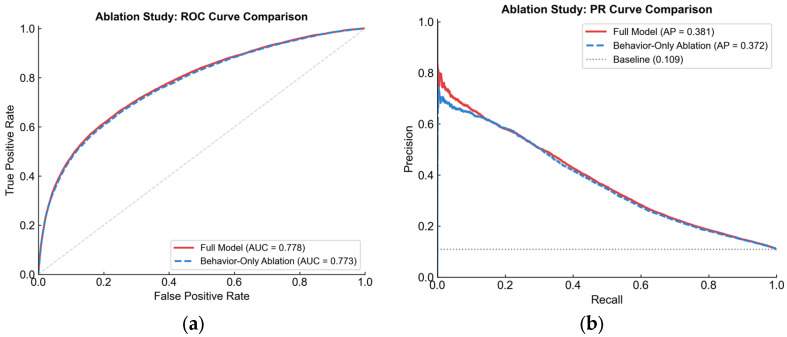
Comparative performance analysis between the full-feature model and the behavior-only ablation model. (**a**) Receiver Operating Characteristic (ROC) curve comparison; (**b**) Precision–Recall (PR) curve comparison. The light blue diagonal dashed line represents the reference line for a random classifier (AUC = 0.5).

To verify whether “behavioral features” possess independent and comprehensive representational capabilities, we designed a rigorous ablation experiment. We completely removed all absolute growth and developmental metrics from the pure behavioral model. The experimental results (shown in Figure 5) indicate that the pure behavioral model, after removing absolute age and body weight, not only did not exhibit a decline in performance but actually performed on par with the full-feature model (AUC: 0.773 vs. 0.778; AP: 0.372 vs. 0.381). This demonstrates that for pigs in commercial farming, their continuous behavioral trait trajectories alone can be used to predict growth impairment, without necessarily relying heavily on static data reflecting the current state, such as current body weight and age. The green dot indicates the optimal decision threshold (0.383) where the F1-score is maximized.

### 3.2. Multi-Scale Feeding Dynamic Signatures Attribution via SHAP

To dismantle the “black box” nature of machine learning and identify the specific behavioral anomalies triggering the warnings, we utilized the SHAP (SHapley Additive exPlanations) framework for a global parsing of the model’s decision-making process.

**Figure 6:**
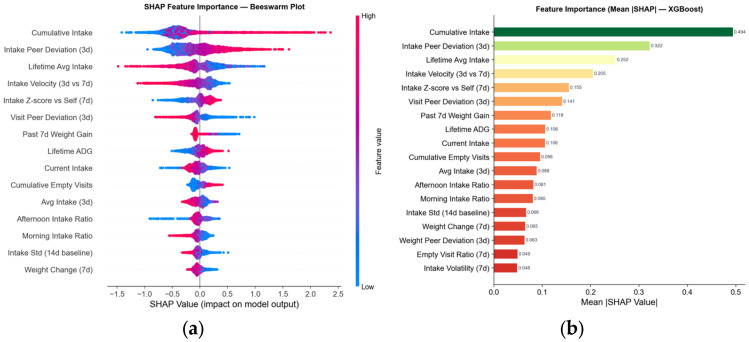
SHAP-based interpretability analysis of the Ensemble model. (**a**) SHAP beeswarm plot; (**b**) SHAP feature importance ranking.

As shown in the SHAP Beeswarm plot (Figure 6a) and the feature importance bar chart (Figure 6b), our refined model has excluded traditional body growth metrics from the core prediction sequence. Instead, it relies on short-term microbehavioral rhythm deviations and long-term cumulative metrics. The top-ranking core drivers in the model importance rankings include:

- Cumulative Intake and Lifetime Avg Intake: These features are consistent with our general expectations, as these metrics form the biological basis of individual body weight.
- Intake Peer Deviation (3d): The model does not focus on evaluating absolute feed intake during this time period but prioritizes the degree of deviation of an individual relative to its peer group. When an individual lags behind its peers, the SHAP value increased sharply.
- Intake Velocity: The kinetic decay trend of feed intake over consecutive days.

The SHAP analysis mechanistically confirms our core hypothesis: in the earliest stages of disease or sub-health, animals first exhibit disruptions in behavioral rhythms (e.g., deviation from age-group norms), followed by the emergence of physical pathological symptoms.

### 3.3. Clinical Translation and Behavioral Profiling: Early Warning Lead Time Distribution

**Figure 7:**
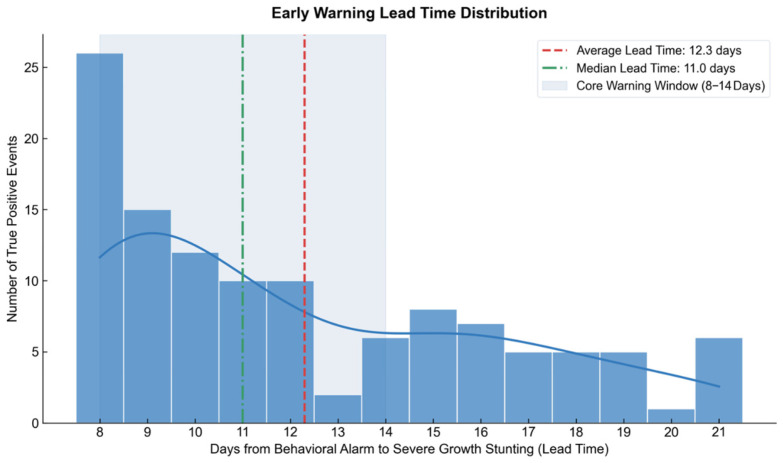
Early Warning Lead Time Distribution. The blue solid line represents the smoothed trend line illustrating the distribution profile.

The direct predictive objective at the foundational level of the model is to determine whether behavioral characteristics preceding the development of “underperforming pigs” will occur within the next few days. However, this “behavioral collapse” is merely the starting point of reduced productivity. In actual swine farming, there is a significant physiological lag between “abnormal behavioral characteristics” and “externally visible severe growth deceleration.” To quantify the practical clinical intervention value of this system, we conducted a retrospective analysis of the “true positive” results from the model’s early warnings and the subsequent actual growth deceleration observed in the predicted subjects. Statistical distributions show that when the system issues an alert based on abnormal behavioral characteristics, the target pigs typically begin to exhibit significant phenotypic growth stagnation within the subsequent 8 to 15 days. Quantitative calculations indicate that this behavior-driven early warning model provides an average lead time of 12.3 days and a median lead time of 11.0 days, with over 65% of early warning events concentrated within the core time window of 8 to 14 days in advance (see Figure 7).

### 3.4. The “Pseudo-Appetite” Mechanism

In the early stages of growth deceleration, although animals’ effective nutrient intake drops sharply, they continue to visit feeding stations frequently without eating, resulting in a high rate of empty visit ratio.

**Figure 8:**
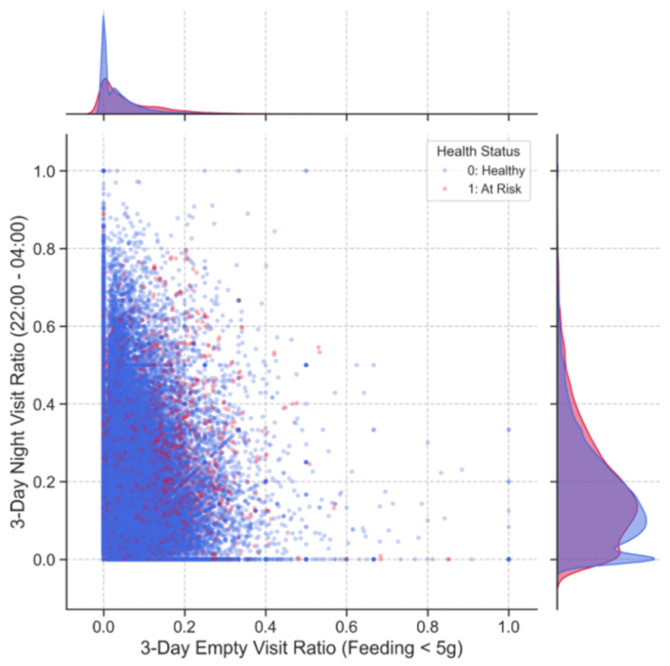
Joint distribution of behavioral features categorized by health status. The central scatter plot illustrates the relationship between the “3-Day Empty Visit Ratio” (*x*-axis) and the “3-Day Night Visit Ratio” (*y*-axis), with individual observations color-coded by health status (Healthy vs. At-Risk). The marginal plots along the top and right axes display the Kernel Density Estimate (KDE) for each feature, highlighting distributional shifts and overlapping characteristics between the healthy and at-risk cohorts, where the blue and red filled areas correspond to the healthy and at-risk groups, respectively.

The Joint Plot illustrates the behavioral divergence between the at-risk group and the healthy cohort as is shown in Figure 8. Specifically, at-risk individuals exhibit a slightly special distribution in the regions of High Empty Visit Ratio (>0.2) and High Night Visit Ratio. This behavioral pattern suggests that although growth-impaired individuals have reached a plateau in weight gain, their frequency of station-visiting remains disproportionately high. This phenomenon, which can be characterized as “pseudo-appetite” hypothesis, reflects a collapse of ingestion efficiency in some situations of “underperforming pigs”: some pigs maintain the occupation of feeding stations without effective nutrient intake.

### 3.5. Diurnal Rhythm Divergence and “Herd Dependency” Logic

**Figure 9:**
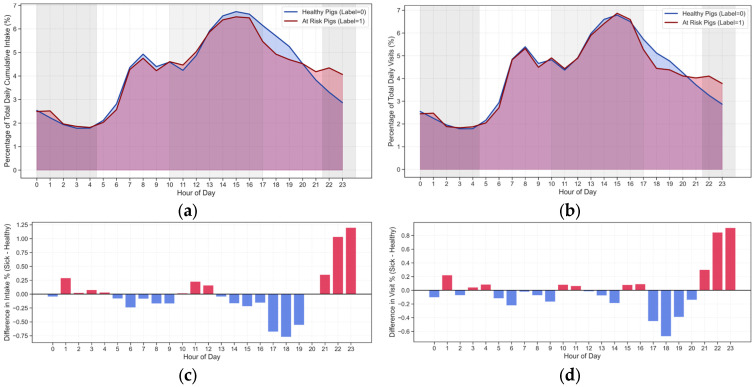
Analysis of diurnal feeding dynamics and behavioral deviations. (**a**) Diurnal distribution of total feed intake; (**b**) Diurnal distribution of station visit frequency; (**c**) Diurnal difference in total feed intake between high-risk and healthy cohorts; (**d**) Diurnal difference in station visit frequency. The red areas in (**c**,**d**) indicate periods when the feed intake of underperforming pigs is higher than that of the healthy control cohort, whereas the blue areas represent the opposite.

To quantify these rhythm disturbances, we plotted the relative differences in feeding frequency and actual intake based on a 24 h cycle (Figure 9a,b).

To examine deviations in diurnal feeding patterns, we compared the overall data from the normal group with those from the high-risk group exhibiting abnormal diurnal feeding habits. The analysis of differences shown in Figure 9c,d indicates that, compared to the healthy group, high-risk (growth-retarded) pigs exhibit significant diurnal deviations in their feeding habits. Specifically, during the typical evening feeding peak (17:00–19:00), the high-risk group exhibited deficiencies in both actual feed intake and feeding frequency (dark blue bars). Conversely, during the late-night period (21:00–23:00), feeding attempts showed a compensatory increase, with the activity levels of high-risk pigs significantly exceeding the baseline of the healthy group (red bars).

This temporal deviation may indicate that high-risk individuals are less competitive for feed than normal pigs. We propose the following hypothesis: due to their compromised physical condition, high-risk pigs are likely to face social marginalization or “bullying” from their healthier peers, which may effectively restrict their access to feeding stations during normal peak periods. Furthermore, this circadian disruption reflects underlying physiological abnormalities. Unable to meet the energy demands required for peak-period competition, these compromised animals are forced to adopt asynchronous feeding strategies—attempting to forage at night when feeders are unoccupied. However, due to impaired feeding or nutrient utilization resulting from compromised physiological function, these late-night feeding behaviors often fail to translate into effective nutrient intake, thereby further exacerbating their growth retardation. This hypothesis can explain certain phenomena, but further quantitative research is needed to verify its validity.

## 4. Discussion

### 4.1. Shifting the Focus from “Weight Prediction” to “Growth Impairment Precursor Detection”

Currently, most research on pig growth focuses on using machine learning for real-time or final weight prediction, which essentially involves fitting models to “post-event states.” However, in production practices, identifying a pig that has already experienced severe weight loss is far less valuable than issuing an early warning before emaciation occurs. The primary contribution of this study lies in redefining the research task from linear fitting of physiological indicators to the early detection of the “risk of growth collapse.” Our results indicate that feeding behavioral patterns themselves possess a certain—and even relatively high—level of predictive capability. However, due to the dataset’s limitation to pure time-series data (excluding photographic or video data), we were unable to extract more multidimensional behavioral feature fingerprints from the pigs. At the same time, due to the presence of compensatory growth, we acknowledge that not all pigs with reduced feed intake will ultimately exhibit significant growth retardation, as some individuals may catch up through compensatory growth. However, the number of times an individual is flagged in this system is also significant: field tests have shown that individuals who are flagged repeatedly or over an extended period almost invariably experience growth impairment, which partially mitigates the issue of false positives. Nevertheless, the accuracy of the proposed model remains acceptable compared to manual screening and can serve as a preliminary screening tool or supplementary early warning system. In our future research, we will further investigate the relationship between foraging behavior and growth, and seek to obtain more comprehensive—and even multimodal—datasets, such as video time-series analysis rather than traditional image analysis, in order to better address some of the current limitations of this study.

### 4.2. The “Pseudo-Appetite” Trap Hypothesis and Ingestion Efficiency Collapse

Traditional swine health monitoring is primarily based on the intuitive assumption of “decreased feed intake.” However, our analysis reveals a counterintuitive phenomenon: during the early high-risk stage of growth retardation, the average daily feed intake (2.46 kg) of some individuals was actually slightly higher than that of the healthy group (2.38 kg). This finding challenges the monitoring paradigm that relies solely on absolute feed intake. We define this phenomenon as the “pseudo-appetite trap” hypothesis. By analyzing dynamic characteristics and behavioral snapshots, we found that the ratio of empty visits surged significantly among high-risk individuals, accompanied by a higher binge-feeding bias. This suggests that “ineffective occupation” or compensatory foraging behavior triggered by physiological discomfort is a major behavioral characteristic of these pigs. Such behavior masks the lack of effective nutrient intake, ultimately leading to a decline in feed conversion ratio (FCR). This demonstrates that behavioral rhythms and frequencies sometimes hold greater predictive value than absolute body weight or feed intake data. However, this could also be due to limitations in the dataset or mere chance. This hypothesis also requires further research to be substantiated.

### 4.3. Social Hierarchy Variation and the “Herd Dependency” Bullying Hypothesis

This study indicates that social competition (bullying) may be a key behavioral precursor to health collapse. Healthy pigs dominate competitive daytime feeding peaks (e.g., 17:00–19:00), reflecting their physical dominance within the social hierarchy. In contrast, individuals in high-risk periods exhibit forced “asynchronous foraging” behavior. Due to declining physical strength and competitive status, they suffer severe social marginalization during normal feeding periods. Behaviorally, this forces them to engage in compensatory foraging at night (e.g., 21:00–23:00), attempting to feed when the feeders are unoccupied.

This behavioral pattern holds significant research value, offering a new perspective on social hierarchy, social exclusion, and competitive behavior in animal ethology. It should be noted that this study was limited by the absence of video and image data, preventing quantitative tracking of this behavior; most conclusions are derived from observations, inferences, and preliminary validation of characteristic data. Future studies with video monitoring capabilities could further validate and expand this behavioral hypothesis.

### 4.4. “Age Decoupling” of Features and In-Field Stage Adaptability

Another key finding of this study is that we successfully achieved desensitization of the model to absolute age. Traditional assessment models heavily rely on target weights at specific ages; this “shortcut learning” often results in poor transferability between different growth stages. Ablation experiments confirmed that, after removing absolute age and weight features, the performance of the model based solely on behavioral features was not substantially affected and remained acceptable. This suggests that relative behavioral deviations and cumulative life-cycle features can serve as the true “first principles” for swine health assessment.

This “age decoupling” characteristic enables the model to demonstrate good internal consistency within the studied environmental context, allowing it to adapt well to different growth stages from early fattening to the market-ready stage. This provides a promising technical foundation for alleviating model overfitting—a long-standing industry pain point in Precision Livestock Farming (PLF) applications. However, it must be noted that since all data in this study were sourced from a single commercial farming enterprise, extensive external validation is still required on independent farms with different management practices, feed formulations, and climatic characteristics to further confirm the framework’s broad generalizability across farms.

## 5. Conclusions

This study constructed an Ensemble-based (combining XGBoost and LightGBM) early health warning pipeline capable of spanning the entire swine life cycle, utilizing massive time-series data from Electronic Feeding Stations (EFS) and innovative “cohort norm comparison” and “unbounded rolling feature extraction” techniques.

The experimental results demonstrate that the model can issue alerts an average of 12.3 days before individuals enter a state of substantial growth impairment. This study not only proposed hypotheses such as the “pseudo-appetite trap” and “group-dependent logic” through SHAP attribution analysis, but also achieved acceptable model performance (ROC-AUC of 0.778 for the full-feature model and 0.773 for the behavioral-feature-only model) as well as cross-stage generalization capability (age decoupling). In terms of accuracy, this study achieved a 3.5-fold improvement over random prediction (from a baseline of 10.9% to 38.16%), providing robust algorithmic support and a theoretical foundation for the paradigm shift in precision livestock farming from “post hoc statistics” to “predictive intervention”.

## Data Availability

The data presented in this study are available on request from the corresponding author. The data are not publicly available due to privacy and ethical restrictions related to the commercial breeding farm’s proprietary information.

## Abbreviations

The following abbreviations are used in this manuscript:

PLF	Precision Livestock Farming
EFS	Electronic Feeding Station
AFS	Automated Feeding Systems
RFID	Radio Frequency Identification
IQR	Interquartile Range
SSE	Sum of Squared Errors
XGBoost	eXtreme Gradient Boosting
AUC	Area Under the Curve
SHAP	SHapley Additive exPlanations
FCR	Feed Conversion Ratio

## Appendix A

### Appendix A.2

**Table A1:** Number statistics for different pigs.

Breed	Number	Percentage
Duroc	2274	37.68%
Landrace	1544	25.58%
Large White	1149	19.04%
Pietrain	1058	17.53%
Pietrain × Duroc	10	0.17%
Total	6035	100%

### Appendix A.3

**Table A2:** Description of variables extracted from the Intelligent Feeding System (IFS).

Variable Name	Scientific Description	Unit
station_id	Unique identifier of the feeding station	-
user_defined_id	Identification code assigned by the farm management	-
rf_id_tag	Radio Frequency Identification tag	-
entry_time/exit_time	Timestamp of entry into and exit from the feeding station	DateTime
feeding_duration_sec	Total duration of a single feeding event	Seconds
feeding_amount	Amount of feed consumed during a single visit	kg
current_weight	Real-time body weight recorded during the visit	kg
entry_weight/exit_weight	Body weight at the start and end of the visit	kg
refill_weight	Weight of feed added to the station hopper	kg
individual_id	Unique identification number for the pig	-
ear_tag	Official ear tag number	-
breed	Genetic breed classification	-
birth_date	Date of birth	Date
gender	Biological sex	-
age	Pig age at the time of the record	Days
